# Temperature limits for storage of extended boar semen from the perspective of the sperm's energy status

**DOI:** 10.3389/fvets.2022.953021

**Published:** 2022-08-05

**Authors:** Heiko Henning, Quynh Thu Nguyen, Ulrike Wallner, Dagmar Waberski

**Affiliations:** ^1^Unit for Reproductive Medicine/Clinic for Pigs and Small Ruminants, University of Veterinary Medicine Hannover, Hannover, Germany; ^2^Friedrich-Loeffler-Institut, Institute of Farm Animal Genetics, Neustadt am Rübenberge, Germany; ^3^Department of Animal Sciences, University of Göttingen, Göttingen, Germany

**Keywords:** spermatozoa, boar semen, storage temperature, chilling, energy metabolism, ATP

## Abstract

The optimum storage temperature for liquid-preserved boar semen has been empirically determined to be between 15 and 20°C. Lower temperatures provide an advantage to inhibit bacterial growth, but are regarded as critical due to the high sensitivity of boar spermatozoa to chilling injury. Higher storage temperatures are supposed to induce energy deficiency due to an insufficient depression of metabolic cell activity. However, experimental evidence for alterations of the sperm's energy status in relation to storage temperature and duration is missing. Therefore, we aimed to revisit the upper and lower storage temperature limits for liquid-preserved boar semen from the perspective of the sperm's energy metabolism. Ejaculates (*n* = 7 boars) were cooled down in Beltsville Thawing Solution (BTS) to 25, 17, 10, or 5°C and stored for up to 120 h. ATP and adenylate energy charge (EC) levels were assessed at storage temperature (24, 72, and 120 h storage) and after subsequent re-warming (38°C). Sperm quality and energy status remained at a stable level in samples stored at 25 and 17°C. Chilling to and storage at 10 or 5°C in BTS provoked cold shock in a subset of sperm as shown by a loss in viability and motility (*P* < 0.05), which was accompanied by a significant release of adenine nucleotides into the semen extender. Prolonged storage for 120 h resulted in significantly lower mean ATP concentrations in viable spermatozoa at 5 or 10°C compared to 17°C (*P* < 0.05). Cluster analysis revealed that the main sperm subpopulation, i.e., sperm with moderate speed and linearity, decreased from 50 to 30% (*P* < 0.05) in favor of slow-moving spermatozoa (5°C) or spermatozoa with a hyperactivation-like motility pattern (10°C). The results point to a sublethal imbalance in available ATP in a subset of the surviving sperm population, rather than a general decrease in available ATP in all spermatozoa. In conclusion, storing diluted boar semen at a stable temperature between 17 and 25°C is a safe procedure concerning the spermatozoa's energy status. Future concepts for hypothermic boar semen preservation below 17°C require measures which ameliorate the imbalanced energy status in viable spermatozoa.

## Introduction

Artificial insemination with liquid preserved semen is the most efficient and widely used assisted reproductive technique in pig breeding (1). The production management of the semen doses, especially the temperature management during semen dilution and chilling, and the final storage temperature of the samples have received much attention and refinement in past years (2–4). In commercial settings, the recommended storage temperature for liquid preserved semen is still 17°C (15–20°C) (1, 5, 6). This temperature range has been empirically determined in studies dating back to the late 1940s and 1950s (7, 8). It is considered as a compromise between preserving sperm longevity by depressing the sperm cell's metabolic activity, whilst not provoking an irreversible loss of selective permeability and integrity of the plasma membrane (9).

The long-standing situation was that chilling and storage of boar spermatozoa results in an inevitable cold shock which comprises their viability (5, 9). The lower temperature limits for boar semen storage have been suggested to be between 10 and 12°C (9, 10). Beyond the increase in antibiotic multi-drug resistance, storage at lower temperature is desired to inhibit bacterial growth. Recently, the suggested lower temperature limits have been successfully shifted toward 5°C without comprising semen quality or fertility (11–14). It is not only that chilling may corrupt the cellular integrity but also the resumption of metabolic activity in viable sperm during rewarming to body temperature after insemination. Such sublethal chilling-related injury becomes especially obvious during warming in the temperature range between 20 and 38°C (15, 16). The impact of a rapid temperature rise is sometimes referred to as warm shock and includes, for example, a rise in free intracellular calcium ion concentrations and capacitation-like changes in viable cells (15–17). The regulation and maintenance of low intracellular free calcium levels in spermatozoa rely on ATP-dependent molecules (18, 19).

In contrast to the temperature range below 15°C, storage temperatures above 20°C are perceived as not fully inhibiting the sperm metabolism, thus leading to a depletion of sperm energy reserves and an accumulation of metabolic (by-)products (9, 20). Therefore, semen storage above 20°C is not desired, but may occur during transport in bulk of freshly filled semen portions in insulated boxes or during the hot season, or when on-farm semen storage units are not functioning properly. On the other hand, available data on viability, motility or acrosome integrity indicate an identical or even better semen quality for samples at 25°C than at 17°C or 15°C (20, 21). To our knowledge, only one publication points to a slightly lower mitochondrial oxidoreductive capability of sperm after four or more days of storage at 25°C when compared to samples stored at 17°C, despite a tendency to a lower percentage of viable spermatozoa with active mitochondria (21). Data on the energy status of boar spermatozoa preserved at 20–25°C are missing. The lack of evidence for a metabolic exhaustion of spermatozoa intentionally or accidentally stored at temperatures above 20°C therefore prompted us to challenge this long-standing dogma.

The aim of the present study was to revisit the current recommendations for the storage temperature of liquid preserved boar semen from the perspective of the sperm energy state. Therefore, the influence of different storage temperatures, i.e., 25, 17, 10, and 5°C, and storage times on ATP levels and adenylate energy charge (EC) was assessed directly after storage and after re-warming of the samples to body temperature. The relation between sperm subpopulations with different motility patterns, the spermatozoa's energy status, and mitochondrial function was explored.

## Materials and methods

### Experimental design

Semen was extended in Beltsville Thawing Solution (BTS) to a concentration of 20 × 10^6^ sperm/mL. Split samples were stored at 25, 17, 10, or 5°C, and analyzed after 24, 72, and 120 h storage. The ATP concentration, EC, as well as viability and acrosome integrity were assessed at storage temperature and after a 30-min incubation period of the samples at 38°C. The percentage of viable spermatozoa with high mitochondrial transmembrane potential as assessed by flow cytometry, and motility parameters as assessed by computer assisted semen analysis (CASA) were only determined in samples incubated for 30 min at 38°C.

### Chemicals

All chemicals were of analytical grade and, unless otherwise stated, obtained from Sigma-Aldrich (Steinheim, Germany), Merck (Darmstadt, Germany), and Roth (Karlsruhe, Germany), respectively. Propidium iodide (PI) was obtained from Sigma Aldrich (Steinheim, Germany), while Hoechst 33342 (H342) was purchased from Life Technologies (Darmstadt, Germany). Peanut agglutinin conjugated to fluorescein-isothiocyanate (PNA-FITC) and 5,5′,6,6′-tetrachloro-1,1′,3,3′-tetraethyl-imidacarbocyanine iodide (JC-1) were both from Enzo Life Sciences (Lörrach, Germany).

### Semen processing

Semen was collected from seven healthy, mature boars housed at the facilities of the Unit for Reproductive Medicine, University of Veterinary Medicine Hannover. Boars were kept and handled in accordance with the European Commission Directive for Pig Welfare. From each boar, one ejaculate was collected by the gloved hand method into disposable collection bags with integrated filter to remove the gel fraction. Collection bags were mounted in pre-warmed thermos cups (38°C) to prevent any cold-shock for the spermatozoa. After collection, the semen was immediately transferred to the laboratory in Styrofoam boxes. The raw semen was evaluated as previously described (22). Only ejaculates with ≥70% motile and ≤25% morphologically abnormal sperm were diluted isothermically (33°C) with BTS extender (Minitüb, Tiefenbach, Germany) to a concentration of 20 × 10^6^ cells/mL. Four samples of 100 mL semen were prepared in screw cap bottles for semen storage, i.e., one bottle for each storage temperature. The samples were cooled stepwise to the desired storage temperature. Samples designed for storage at 25°C were kept for 60 min at room temperature (RT; 20 to 22°C) and then transferred to an incubator set at 25°C. Samples designed for storage at 17°C were kept for 90 min at RT and then placed in a 17°C storage unit. Samples prepared for 10°C storage were kept for 90 min at RT, then for 60 min at 17°C before being placed in a 10°C storage unit. Samples designed for 5°C storage were kept for 90 min at RT, then for 60 min at 17°C, followed by 60 min at 10°C, and then transferred to a 5°C storage unit. All samples were stored in the dark.

### Computer-assisted semen analysis (CASA)

Semen samples were analyzed on a computer-assisted sperm analysis system (SpermVision^®^ version 3.5; Minitüb, Tiefenbach, Germany) as previously described (23). Briefly, 2 mL of semen was incubated at 38°C for 30 min before motility was analyzed using four-chamber slides (Leja, Nieuw Vennep, The Netherlands) with a chamber depth of 20 μm. Total motility and progressive motility were recorded as well as average path velocity (VAP, μm/s), curvilinear velocity (VCL, μm/s), straight-line velocity (VSL; μm/s), straightness (STR = VSL/VAP), linearity (LIN = VSL/VCL), wobble (WOB = VAP/VCL), average amplitude of lateral head-displacement (ALH; μm), and beat cross frequency (BCF; Hz) for each single spermatozoon. A spermatozoon was defined by a head area between 23 and 120 μm^2^. It was considered to be motile when its average head orientation change (AOC) was higher than 2.5°, and considered to be progressively motile when the straight-line distance (DSL) exceeded 4.5 μm.

### Assessment of viability and acrosome integrity

The integrity of the plasma and acrosomal membrane was assessed by flow cytometry using a triple stain procedure with propidium iodide (PI), fluorescein-isothiocyanate-conjugated peanut agglutinin (PNA-FITC), and Hoechst 33342 as previously described (22, 23). Briefly, a 5-μL subsample of diluted semen was transferred to 995 μL HEPES-buffered saline medium containing propidium iodide (PI; final concentration 5 μg/mL), FITC-conjugated peanut agglutinin (PNA-FITC; final concentration 3.0 μg/mL), and Hoechst 33342 (final concentration 0.75 μg/mL). The percentage of viable spermatozoa with an intact outer acrosomal membrane (PI and PNA-FITC negative) after either a 15 min incubation period at 20°C or a 30 min incubation period at 38°C was determined in 10,000 events using FloMax v2.4 software (Partec). The former was carried out to exert as little temperature stress as possible on the samples before analysis, without provoking capacitation-like changes (16).

### Assessment of mitochondrial transmembrane potential in viable spermatozoa

The mitochondrial transmembrane potential (MMP) in spermatozoa was assessed by flow cytometry using 5,5′,6,6′-tetrachloro-1,1′,3,3′-tetraethyl-imidacarbocyanine iodide (JC-1) in combination with propidium iodide (PI) and Hoechst 33342 (H342) as described elsewhere (23). Briefly, semen subsamples were stained with Hoechst 33324 (final concentration 2.4 μM), PI (final concentration 30 μM), and the mitochondrial probe JC-1 (final concentration 1.5 μM) and then incubated for 30 min at 38 °C. Viable, i.e., PI negative, spermatozoa with low green, but high orange fluorescence intensity were considered as having a high mitochondrial transmembrane potential (hMMP). Samples stored at 17°C for 24 h served as internal reference to set the threshold between high and low mitochondrial membrane potential.

### ATP and EC assay

The ATP, ADP, and AMP concentrations were determined using a luciferase reaction kit according to Nguyen et al. (22). Samples of diluted boar semen (100 μL) were directly taken at the respective storage temperature (25, 17, 10, or 5°C) and after incubation for 30 min at 38°C. Sperm-free samples of the semen extender were obtained after centrifugation. Samples were mixed with 1 μL phosphatase inhibitor cocktail (P5726, Sigma-Aldrich, Steinheim, Germany) and kept on ice for 30 min. After inhibitor treatment, samples were stored at −20°C for later ATP, ADP, AMP, and EC assessment. Nucleotide extraction was achieved by incubating samples with a boiling buffer (50 mM Tricine, 10 mM MgSO_4_, 2 mM EDTA, pH = 7.8) for 10 min at 95°C. Subsequently, samples were chilled on ice (10 min), followed by centrifugation at 5,000 × g for 30 min at 4°C. The supernatant was used for nucleotide quantification. ATP was directly assessed with a firefly luciferin-luciferase assay (FL-AA, Sigma- Aldrich). ADP and AMP were quantified after stepwise conversion to ATP. Serial dilutions of an ATP standard solution (FLAAS, Sigma Aldrich) served as reference samples for the luciferin-luciferase assay. Light production of ATP standards and samples was assessed with a GENios Pro plate reader (Tecan Group Ltd., Männedorf, Switzerland). ATP concentration of the samples was calculated as pmol ATP per 10^5^ spermatozoa. The EC was calculated as described by Ball and Atkinson (24):


(1) $$\begin{array}{l} Adenylate\;energy\;charge(EC)\\ \;=([ATP]+0.5[ADP])/([ATP]+[ADP]+[AMP]) \end{array}$$

Based on the assumption that only viable spermatozoa, i.e., with an intact plasma membrane, contain a considerable amount of ATP (25), the average ATP content for each viable, acrosome-intact spermatozoon was calculated;


(2) $$\begin{array}{l} ATP{\left(fmol\right)}_{VAIspermatozoon}\\ \;=\frac{ATP{\left(pmol\right)}_{sample}}{100,000spermatozoa^{*}(\frac{\%VAIspermatozoa}{100})}*\;1,000 \end{array}$$

where VAI = viable, acrosome intact.

### Statistical analysis

Data were analyzed with Excel (Microsoft Office 2010, Microsoft Corporation, WA, USA) and Statistical Analysis Software (SAS, version 9.4, Cary, NC, USA). Data from all parameters were tested for normal distribution with a Shapiro Wilk test. A possible effect of storage temperature or storage time on selected parameters was evaluated with the Friedman test. Subsequently, data from different storage temperatures or storage times were compared with Wilcoxon's signed rank test for paired samples. Single sperm data from CASA were subjected to hierarchical cluster analysis (26). Motility descriptors for every single, motile spermatozoon after incubation at 38°C from all four storage temperatures (5, 10, 17, 25°C) and all storage times (24, 72, 120 h) were combined in one data set. Pearson's chi-squared test was used to determine whether distribution of sperm to the different clusters significantly changed with storage temperature or time. Cramer's V (range 0 to 1) was used as a measure of the effect size that temperature or storage time had on the distribution of sperm to the different clusters. For interpretation, the following guidelines were followed as suggested by Cohen (27): V <0.10 = no effect, 0.10 < V ≤ 0.30 = small effect, 0.30 < V ≤ 0.50 = moderate effect, V > 0.50 = strong effect. Spearman rank correlation coefficients between ATP, EC, and selected CASA and flow cytometry parameters were calculated. The significance level was set at *P* < 0.05.

## Results

### ATP content, EC, and membrane integrity of boar sperm at different storage temperatures

**Figure 1:**
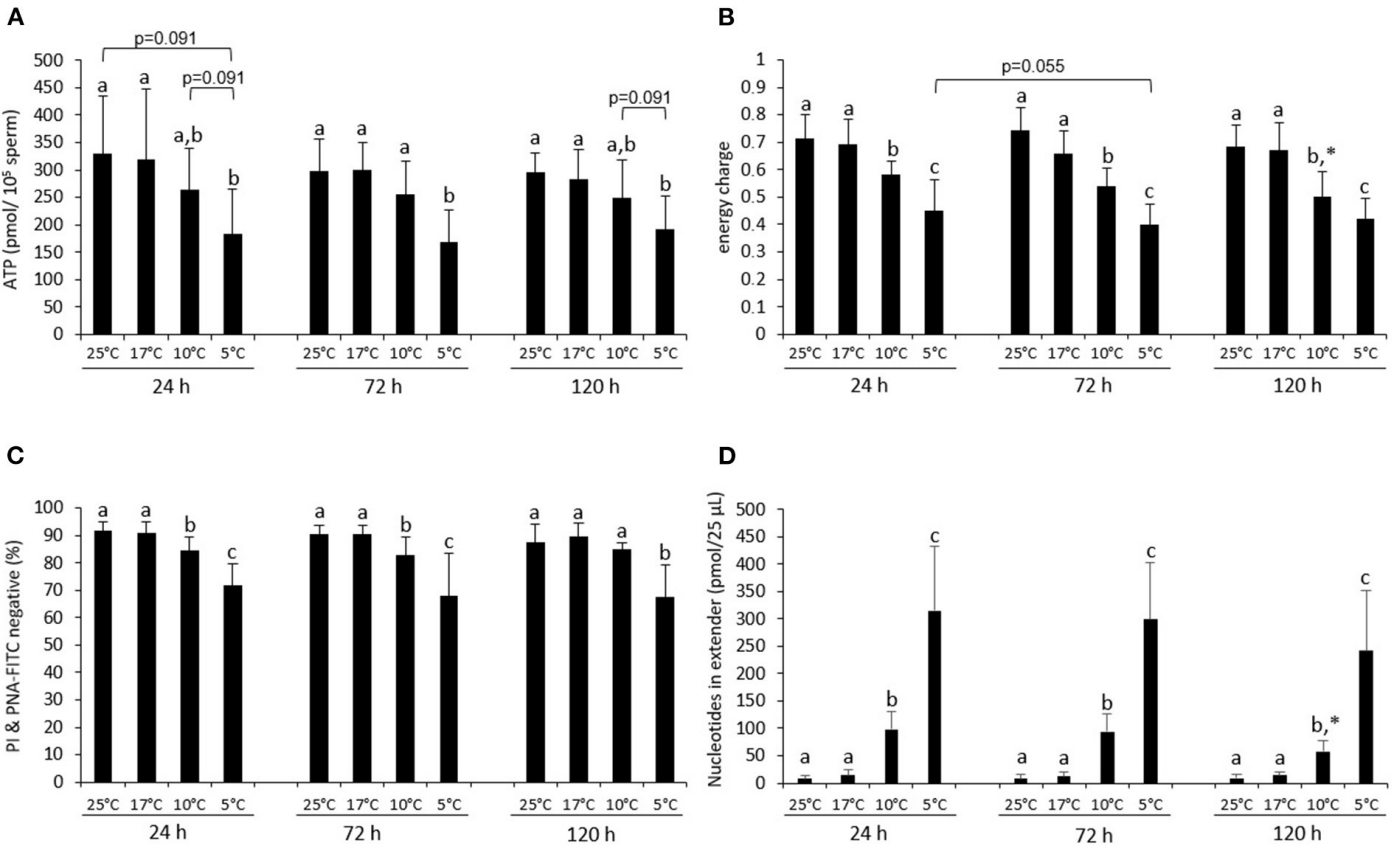
Energy status and sperm viability during storage at different temperatures. ATP content **(A)**, energy charge **(B)**, percentage viable, acrosome-intact sperm [PI & PNA-FITC negative; **(C)**], and sum of nucleotides (ATP + ADP + AMP) in the semen extender **(D)** of boar semen samples stored at various temperatures in Beltsville Thawing Solution. Samples were analyzed directly at the respective storage temperature or after 15 min incubation at RT (PI & PNA-FITC staining). Data are presented as means ± standard deviation (*n* = 7 boars). (a–c) Different letters indicate significant differences between storage temperatures within a given day (*P* < 0.05). An asterisk (*) indicates a significant difference between values after 72 or 120 h storage and the value after 24 h storage (*P* < 0.05).

Values for samples stored at 17°C were considered as reference point, because this temperature has been accepted as optimal storage temperature for liquid preserved pig spermatozoa (1, 5, 6). The ATP content of semen samples at 5°C was on all days of storage lower than for samples at 17°C (Figure 1A). The ATP content of the individual samples did not differ between semen stored at 25, 17, and 10°C at any day of storage. Nonetheless, a significant influence of storage temperature on the calculated average ATP concentration in each viable, acrosome-intact spermatozoon was noted [$\chi_{\left(3\right)}^{2}$ = 11.114, *P* = 0.011; Supplementary Figure 1A].

The average EC did not differ between samples stored at 17 or 25°C and was on all days higher than 0.65 (Figure 1B). At all days, samples stored at 10 or 5°C had a lower EC when compared to samples stored at 17 and 25°C. The EC in samples stored at 5°C was lower than the EC for samples at 10°C.

The percentage of viable sperm with intact acrosomes (PI & PNA-FITC negative) was on average higher than 65% at all storage temperatures and times (Figure 1C). Samples stored at 25 or 17°C did not differ at any day of storage. Samples stored at 10 or 5°C contained fewer viable, acrosome-intact sperm than samples stored at 17 or 25°C at 24 h and 72 h (*P* < 0.05). At 120 h, only samples at 5°C differed from the samples at 17 and 25°C (both *P* < 0.05). At all times, more viable, acrosome-intact spermatozoa were present in samples preserved at 10°C than at 5°C (Figure 1C).

The sum of nucleotides (AMP + ADP + ATP) detected in the semen extender was negligible for samples stored at 25 and 17°C at any time of storage (Figure 1D). However, the sum of free nucleotides in the semen extender increased 7-fold and 22.5-fold in samples chilled to 10 or 5°C, respectively (*P* < 0.05; Figure 1D). The predominant nucleotide in the extender was AMP (Supplementary Table 1).

### ATP content, EC, mitochondrial transmembrane potential and membrane integrity after incubation at 38°C

**Figure 2:**
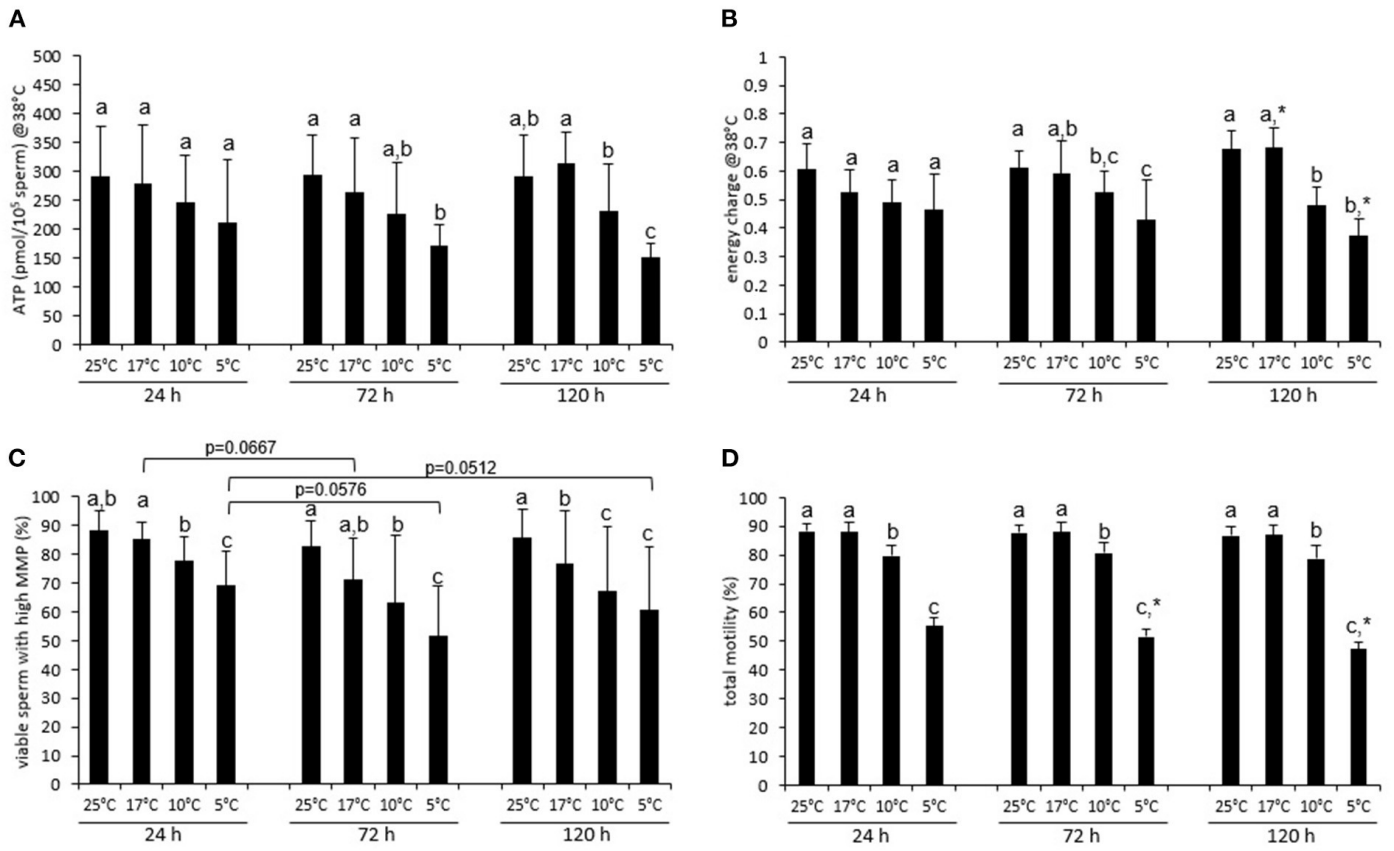
Energy status, mitochondrial function and motility of stored spermatozoa after incubation at 38°C. ATP content **(A)**, energy charge **(B)**, percentage of viable sperm with high mitochondrial transmembrane potential [hMMP; **(C)**], and total motility **(D)** after 30 min incubation at 38°C for extended boar semen samples stored at various temperatures. Data are presented as means ± standard deviation (*n* = 7 boars). (a–c) Different letters indicate significant differences between storage temperatures within a given day (*P* <0 .05). An asterisk (*) indicates a significant difference between values after 72 or 120 h storage and the value after 24 h storage (*P* < 0.05). Values before incubation are presented in Figure 1.

The ATP content of the semen samples after incubation at 38°C was at 24 h similar, irrespective of the storage temperature (Figure 2A; *P* > 0.05). After 72 h and 120 h storage at 5°C, samples had a lower ATP content than the samples stored at 17°C. The ATP levels for samples stored at 10°C were lower compared to samples stored at 17°C only after 120 h storage. Only at this time was the calculated average ATP content for each viable, acrosome-intact spermatozoon significantly lower in samples that had been stored at 5 or 10°C compared to those stored at 17 or 25°C (*P* < 0.05; Supplementary Figure 1B). Samples preserved at 25 and 17°C did not differ at any storage time concerning the average or single cell level (*P* > 0.05; Figure 2A; Supplementary Figure 1B).

The EC after incubation at 38°C was lower than at the respective storage temperature for samples that had been preserved for 24 h at 25°C (Δ = 0.11 ± 0.09), 17°C (Δ = 0.16 ± 0.13), or 10°C (Δ = 0.09 ± 0.09, *P* < 0.05, c.f. Figures 1B, 2B). After 72 h, a significant decline in EC upon incubation at 38°C was only evident for samples that had been preserved at 25°C (Δ = 0.13 ± 0.09, *P* < 0.05). After 120 h, a decline in EC upon incubation was absent. Noteworthy, the already low EC in samples stored at 5°C did not significantly change upon incubation at 38°C (*P* > 0.05). There were no differences in EC after incubation at 38°C for samples stored at the different temperatures for 24 h (Figure 2B). At 72 and 120 h, samples stored at 5°C had a lower EC compared to those stored at 17°C. At 120 h, also samples stored at 10°C (0.50 ± 0.09) had a distinctly lower EC compared to those stored at 17°C (0.68 ± 0.10; *P* < 0.05).

The percentage of viable spermatozoa with hMMP was regularly the highest in samples stored at 25 or 17°C (Figure 2C). At 24 h, the percentage of viable sperm with hMMP was lower, the colder the spermatozoa had been stored (17°C: 85.3 ± 5.9%, 10°C: 77.4 ± 8.8%, 5°C: 69.2 ± 12.1%; all *P* < 0.05). The gradual differences between storage temperatures became more evident the longer semen was stored. The highest percentage of viable sperm with hMMP was maintained in samples stored at 25°C (Figure 2C).

The percentage of viable, acrosome-intact spermatozoa after incubation at 38°C was on average higher than 80% for all storage temperature and time combinations, except for samples stored at 5°C (24 h: 70.2 ± 8.1%, 120 h: 67.2 ± 12.1%; Supplementary Figure 2). Samples stored at 25°C did not differ from those stored at 17°C. Samples stored at 17°C had higher values for viable, acrosome- intact spermatozoa than samples stored at 10°C.

### Motility and kinematic patterns of samples stored at different temperatures

The average motility (total) was higher than 75% for samples stored up to 120 h at 25, 17, or 10°C (Figure 2D, Supplementary Table 2). However, motility was consistently lower for samples stored at 10°C than for those stored at 17 or 25°C (both *P* < 0.05). Semen stored at 5°C contained always the lowest number of motile spermatozoa (<60%) (Figure 2D, Supplementary Table 2). A small, albeit significant decrease in motility over time was only noted for samples stored at 5°C [$\chi_{\left(2\right)}^{2}$ = 14.000, *P* = 0.001, Figure 2D].

**Figure 3:**
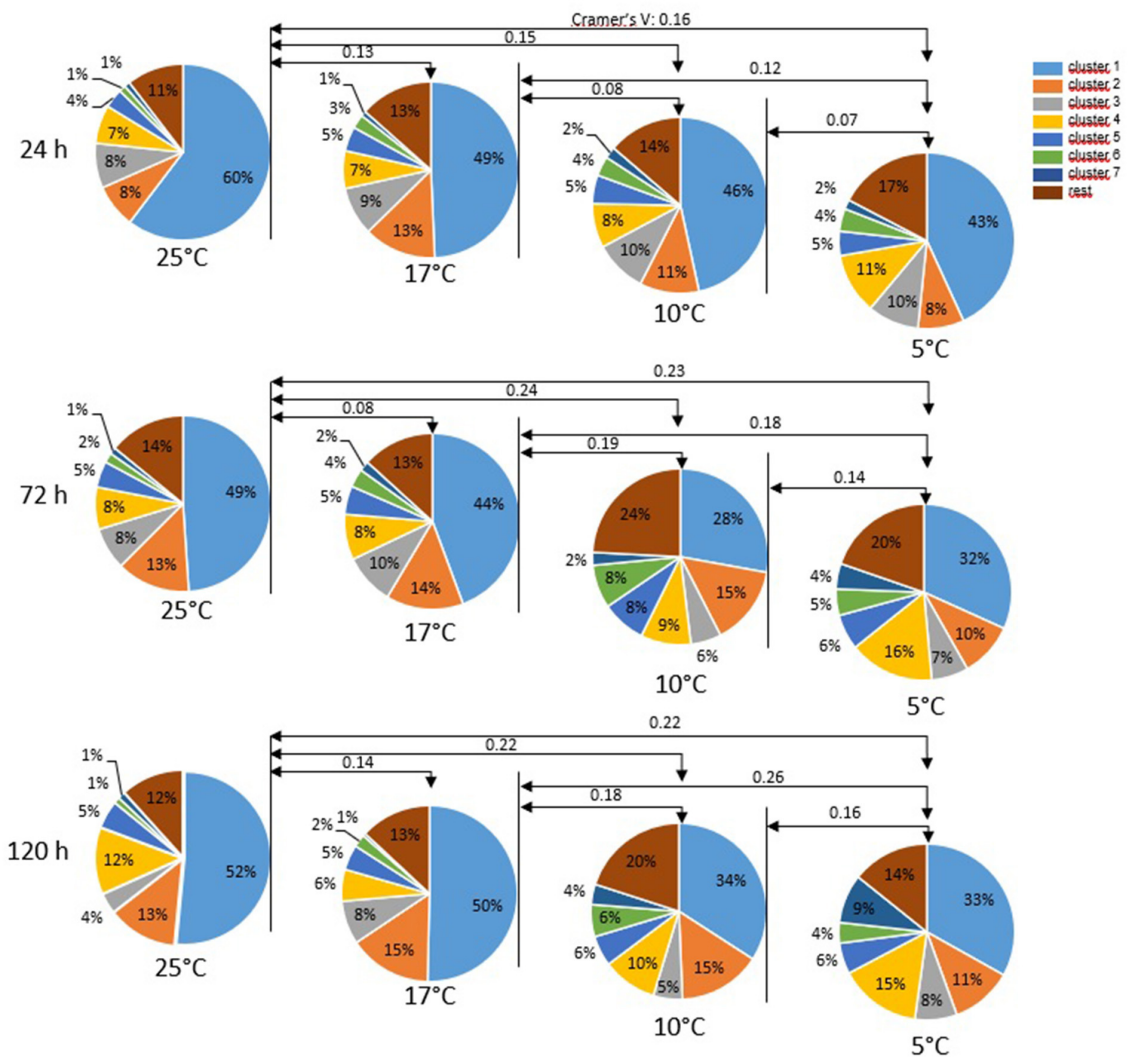
Motility patterns (cluster) of sperm after different storage times at different temperatures. Single sperm data derived from computer-assisted semen analysis (CASA; *n* = 33,229 spermatozoa) were subjected to cluster analysis. Data from all semen storage temperatures and times were combined. Cluster analysis provided a solution with seven main clusters, which explains 68.9 % of the variance in the dataset. Only clusters containing at least at any one time by temperature combination 5% of the spermatozoa are depicted. For motility characteristics of the individual cluster, (see Table 1). Values on arrow lines present Cramer's V which indicates the effect size of storage temperature: V < 0.10 = no effect, 0.10 < V ≤ 0.30 = small effect, 0.30 < V ≤ 0.50 = moderate effect, V > 0.50 = strong effect.

**Figure 4:**
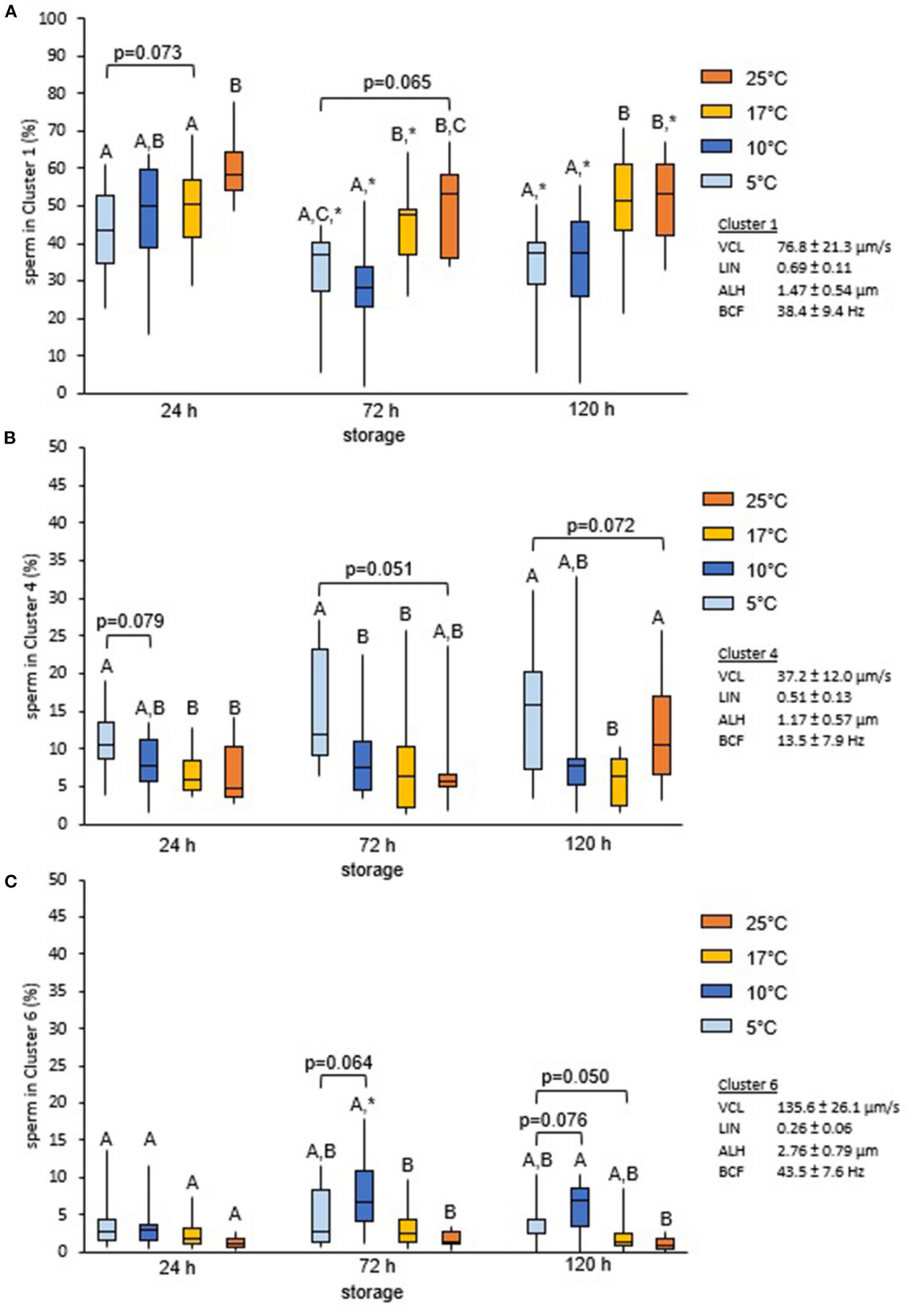
Change in motile sperm subpopulations with storage times and temperature. Change in size of selected cluster in relation to semen storage temperature and time. Spermatozoa were stored in Beltsville Thawing Solution at 25, 17, 10, or 5°C for 24, 72, and 120 h (*n* = 7 boars). Results represent data for Cluster 1 **(A)**, Cluster 4 **(B)**, and Cluster 6 **(C)**. Different capital letters indicate significant differences between storage temperatures at a given storage time (*P* < 0.05). An asterisk (*) indicates a significant change after 72 or 120 h storage as compared to 24 h for a given storage temperature (*P* < 0.05).

Cluster analysis of all motile spermatozoa (*n* = 33,229) provided a solution with seven main clusters which explained 68.9% of the variance in the dataset (Table 1). Storage temperature had a significant impact on sperm distribution to the different clusters, but the effect size for all temperatures, as judged by Cramer's V, was small (Figure 3). Nonetheless, the number of spermatozoa assigned to the major cluster number 1, i.e., sperm with moderate VCL, ALH, and BCF, was lower for samples stored at 5°C than for those stored at 17°C (Figure 4A). Moreover, this cluster became smaller after 72 h and 120 h storage for samples stored at 5 or 10°C, respectively. Concomitantly, samples at 5°C contained at all storage times more sperm with low VCL, ALH, and BCF, i.e., Cluster 4 (Figure 4B) and Cluster 7 (data not shown). Storage at 10°C did not result in an increased number of spermatozoa in Clusters 4 or 7, but in more spermatozoa with hyperactivation-like motility, i.e., spermatozoa in Cluster 6 with high VCL, low LIN, wide ALH, and high BCF. After 72 h, this population was more abundant in samples stored at 10°C than in those stored at 17°C (*P* < 0.05, Figure 4C). A similar trend was detected after 120 h storage for samples stored at 5°C (*P* = 0.050, Figure 4C). At the other end of the tested temperature spectrum, a storage temperature of 25°C resulted in a highly uniform movement pattern in spermatozoa after 24 h, as indicated by 60% spermatozoa in Cluster 1 (Figures 3, 4A). This sperm population decreased significantly only after 120 h and was still equal in size when compared to samples stored at 17°C (*P* > 0.05, Figure 4A). Despite a few significant changes in the size of the sperm cluster with increasing storage time, the overall effect of storage time on sperm motility patterns at the different temperature levels remained small (data not shown).

**Table 1:** Motility characteristics of the different sperm subpopulations.

	**Motility characteristics of sperm**				
	**within a cluster**				
**Cluster**.	**VCL [**μ**m/s]**	**LIN**	**ALH [**μ**m]**	**BCF [Hz]**	**% Sperm**
**no**.		
1	76.8 ± 21.3	0.69 ± 0.11	1.47 ± 0.54	38.4 ± 9.4	44.2
2	128.0 ± 25.4	0.46 ± 0.09	2.74 ± 0.84	44.2 ± 7.8	13.5
3	137.0 ± 29.3	0.76 ± 0.11	2.20 ± 0.83	44.4 ± 7.5	7.9
4	37.2 ± 12.0	0.51 ± 0.13	1.17 ± 0.57	13.5 ± 7.9	8.5
5	76.9 ± 21.5	0.40 ± 0.08	2.14 ± 0.76	36.2 ± 8.6	5.3
6	135.6 ± 26.1	0.26 ± 0.06	2.76 ± 0.79	43.5 ± 7.6	3.4
7	19.8 ± 7.8	0.84 ± 0.11	0.75 ± 0.67	0.5 ± 1.8	1.8
Rest					15.5

Subpopulations of sperm (cluster) with different motility characteristics as detected through cluster analysis. The analysis was based on 33,229 sperm tracks after a 30-min incubation period at 38°C after 24, 72, and 120 h of storage. Only clusters containing more than 5 % of spermatozoa at least at one of the storage times by temperature combinations are listed (c.f. Figure 3). Motility descriptors (mean ± standard deviation) are given for each cluster as well as the percentage of sperm assigned to each cluster.

VCL, curvilinear velocity; LIN, linearity (VSL/VCL); ALH, average amplitude of lateral head-displacement; BCF, beat cross frequency.

### Correlation of energy metabolism with sperm function and integrity

**Figure 5:**
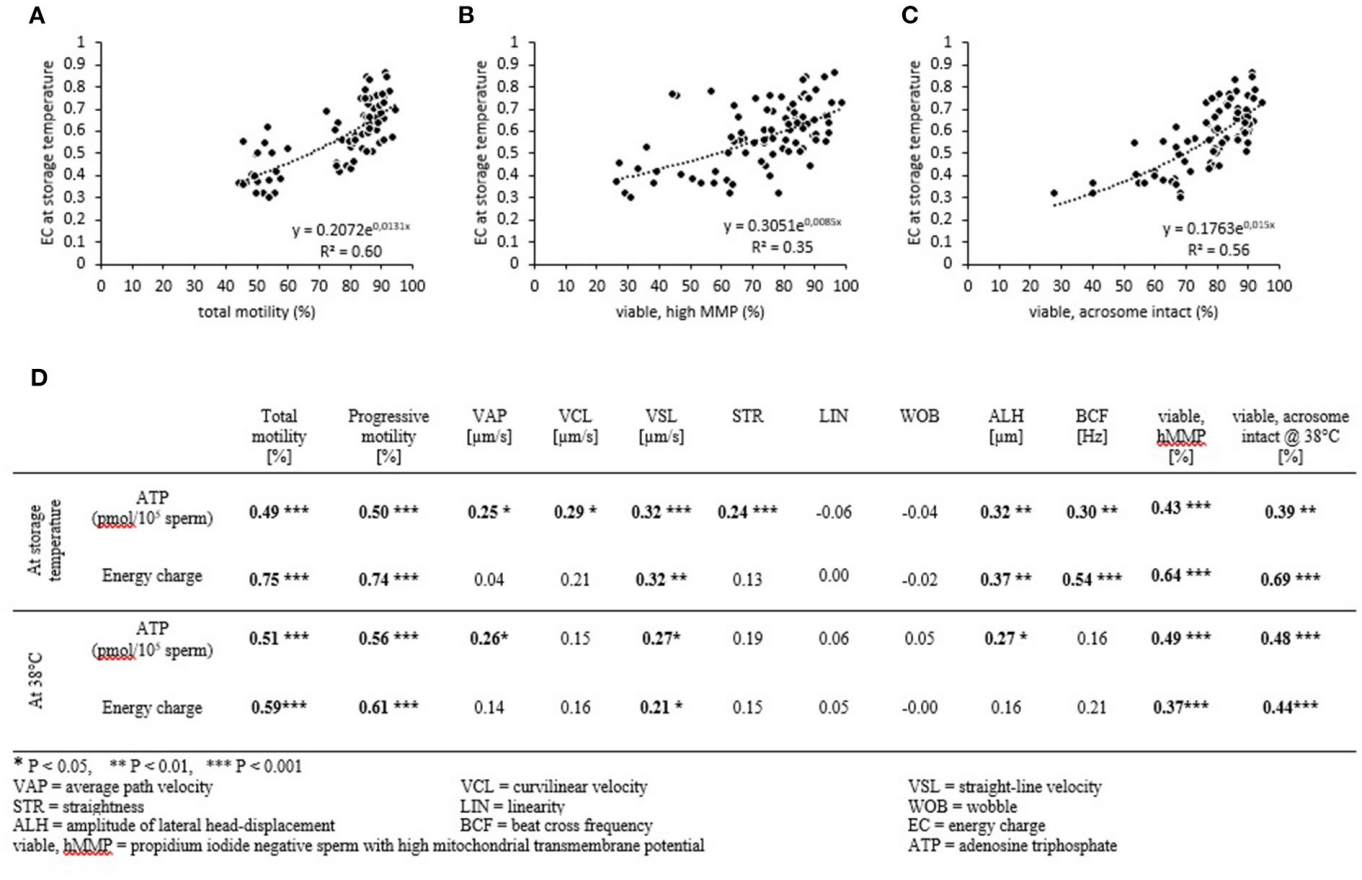
Correlation between energy status, mitochondrial function, and sperm motility. Spearman correlation coefficients for energy charge **(A–D)** and ATP content **(D)** with selected sperm parameters. Samples were assessed after storage **(A–C)** and after incubation at 38°C for 30 min **(D)**. Data from all storage temperatures and times were combined (*n* = 84 samples).

The EC of samples at the given storage temperature was significantly correlated with total motility (*r* = 0.70; *P* < 0.001, Figure 5A), progressive motility (*r* = 0.71; *P* <0 .001), the percentage of viable spermatozoa with hMMP (*r* = 0.49; *P* < 0.001, Figure 5B), and the percentage of viable, acrosome-intact cells at 38°C (*r* = 0.62; *P* < 0.001, Figure 5C). Likewise, the ATP content of samples at the given storage temperature correlated with the aforementioned parameters, but all correlations coefficients were lower (0.23–0.42; Figure 5D). Notably, all samples that reached an EC of ≤0.40 during storage (at 5°C) had a motility of <60% after re-warming. The EC and ATP levels measured at the given storage temperature were also weak to moderate correlated to descriptors of the sperm motility trajectory.

The correlations between EC after incubation at 38°C and total motility (*r* = 0.58; *P* < 0.001), progressive motility (*r* = 0.59; *P* < 0.001), the percentage of viable spermatozoa with hMMP (*r* = 0.39; *P* <0 .001), and the percentage of viable, acrosome-intact cells at 38°C (*r* = 0.20; *P* < 0.01) were weaker than for EC at the storage temperature. The correlations between ATP levels after incubation at 38°C and total motility, progressive motility, the percentage of viable spermatozoa with hMMP, and the percentage of viable acrosome-intact cells at 38°C were similar to those for ATP levels measured at the given storage temperature (Figure 5D).

## Discussion

This study renews the current perception of storage temperature limits for boar semen. It is shown that the empirically determined optimum storage temperature of 15–20°C (5, 8) can be extended to 15–25°C without affecting the energy status, motility, and viability of boar spermatozoa. The current data clearly demonstrate that the potential risk of higher sperm activity and exhaustion due to an increased consumption of energy cannot be confirmed. Neither ATP levels, nor energy charge nor kinematic patterns differed between samples stored at 17 or 25°C. In contrast, the percentage of viable cells with a high mitochondrial transmembrane potential was even slightly better preserved at 25°C and coincided with a large group of sperm with a uniform motility pattern after re-warming to body temperature. Prolonged storage above 25°C, however, needs to consider a higher risk of bacterial growth unless effective antimicrobial control is provided.

A major aim of this study was to elucidate the alteration of the energy metabolism induced by long-term storage at different temperatures and possible effects on sperm survival and motility. Storage at 17°C, which is routinely utilized for boar semen portions, resulted in stable sperm quality and maintained high ATP and EC levels for 5 days. This is consistent with several previous observations (22, 23, 28). Other studies which report a decline in ATP levels during storage, typically also noted a decline in viability or motility (29, 30). This seems logical because gross changes in ATP or EC at the level of a given sample reflect to a large extent the number of viable cells in the sample (31). With the present study, this general perception is supported by the correlations between the EC and, to a lesser extent, ATP concentrations with viability and motility both after storage and after subsequent re-warming.

The effects of energy imbalances on vital sperm parameters became more evident in cold-shocked semen samples. As expected, chilling and storage in the simple extender medium BTS induced loss of sperm motility and viability. The decreased sperm quality was accompanied by loss of ATP and EC in the semen samples together with the detection of nucleotides in the semen extender, thus mirroring the well-known phenomenon of membrane leakage in cold-shocked spermatozoa (32, 33). The present study additionally examined the sperm's ability to restitute their energy metabolism at re-warming to body temperature, an essential step for their journey through the female genital tract after insemination. As shown recently, with ongoing storage time up to seven days, sperm need to invest an increasing amount of ATP to reactivate their motility (23). Thus, the loss of ATP in spermatozoa that experienced sublethal cold shock could be decisive to provide sufficient energy for reactivation of an orchestrated sperm motility after storage, as indicated by the correlation of ATP and EC levels after storage with motility after subsequent rewarming. Interestingly, although the average ATP levels after 24 h storage and re-warming did not differ anymore between samples stored at 5 and 17°C, cluster analysis revealed that the proportion of slow-moving sperm with low vigor of flagellar beating was increased after cold storage. This suggests that a subpopulation of the chilled spermatozoa may have either considerably lower ATP levels, or cannot utilize the ATP as efficiently as others, or have experienced other facets of chilling injury that interfere with motility. The lower amount of viable sperm with hMMP in re-warmed samples previously stored at 5°C compared to the higher storage temperatures could be causative for the observed energetic imbalance. Deficiency in cold storage-induced resumption of energy metabolism becomes more obvious with ongoing storage times as seen by reduced ATP and EC levels being accompanied by a drop in the major sperm subpopulation with average speed, linearity, and vigor to about 32% after 72 h storage. It is to note that boar spermatozoa are extremely sensitive to cold shock damage. Nonetheless, a controlled cooling (34) and a protective extender medium (11, 35, 36) can minimize chilling injury, probably also by maintaining an adequate energy status. The present data suggest that energy charge values below 0.4 are incompatible with the maintenance of a sufficiently high rate of sperm survival in stored semen samples.

While signs of a chilling injury in subsets of viable spermatozoa were readily identified after 24 h at 5°C, storage at 10°C produced a delayed and qualitatively different effect. The proportion of sperm in the major sperm cluster declined from 46% at 24 h to about 28% after 72 h, with a concomitant increased number of spermatozoa with hyperactivation-like motility patterns. At the same time, a high diversification of motility patterns outside the seven major sperm clusters became obvious. Despite these changes, a decline in the calculated average ATP concentrations for each viable spermatozoon was not apparent. Although we cannot exclude that highly diverse energy states exist in the viable sperm population, our observation suggests that other mechanisms than the energy imbalances are involved in the alteration of motility patterns after moderate chilling. This could include a chilling-induced release of calcium from intracellular stores during rewarming (10, 16, 17), which triggers a transient hyperactivation-like motility pattern in a subpopulation of spermatozoa (17, 23). In any case, the data presented here confirm the notion that also from the perspective of energy status, storage at 10°C is inappropriate for boar semen, even if it is only short-term for 24 h.

In conclusion, storing extended boar semen samples at a stable temperature between 17 and 25°C is a safe procedure from the perspective of the spermatozoa's energy status. Chilled storage at 10 and 5°C in a simple, non-protective extender medium provokes disturbed energy balance together with loss of sperm quality. Current developments of low-temperature concepts for boar semen storage should consider maintenance of the energy status during storage and rewarming to body temperature.

## Data availability statement

The original contributions presented in the study are included in the article/Supplementary material, further inquiries can be directed to the corresponding author/s.

## Ethics statement

The animal study was reviewed and approved by the Institutional Animal Welfare Committee at the University of Veterinary Medicine Hannover, Hannover, Germany.

## Author contributions

HH: conceptualization, methodology, formal analysis, supervision, writing—original draft, review, and editing. QN: data curation, formal analysis, writing—original draft. UW: data curation and formal analysis. DW: conceptualization, supervision, funding acquisition, writing—review, and editing. All authors approved the final version of the manuscript.

## Funding

This study was funded by the Lotus-Erasmus Mundus Action 2 (QTN) and the Association for Bioeconomy Research (FBF e.V., Bonn). This Open Access publication was funded by the Deutsche Forschungsgemeinschaft (DFG, German Research Foundation) - 491094227 Open Access Publication Costs and the University of Veterinary Medicine Hannover, Foundation.

## Conflict of interest

The authors declare that the research was conducted in the absence of any commercial or financial relationships that could be construed as a potential conflict of interest.

## Publisher's note

All claims expressed in this article are solely those of the authors and do not necessarily represent those of their affiliated organizations, or those of the publisher, the editors and the reviewers. Any product that may be evaluated in this article, or claim that may be made by its manufacturer, is not guaranteed or endorsed by the publisher.
