# Supplementary material for: Temperature limits for storage of extended boar semen from the perspective of the sperm's energy status
Source: Front Vet Sci. 2022 Aug 5;9:953021. doi: 10.3389/fvets.2022.953021 (PMC9388907; doi:10.3389/fvets.2022.953021)
Supplement: Supplementary file 1 [file Data_Sheet_1.zip › Supplemental Figure 1.docx]

**A**

25°C

17°C

10°C

5°C

storage

120 h

24 h

72 h

**B**

25°C

17°C

10°C

5°C

24 h

72 h

120 h

storage

**Supplemental Figure 1.** Calculated average ATP content in each viable spermatozoon.

Based on the assumption that only viable spermatozoa, i.e. with an intact plasma membrane, contain a considerable amount of ATP, the average ATP content for each viable, acrosome intact spermatozoon was calculated. Spermatozoa were stored in Beltsville Thawing Solution at 25°C, 17°C, 10°C or 5°C for 24 h, 72 h and 120 h (n=7 boars). Results represent data for ATP content A) after storage at various temperatures and B) after subsequent incubation at 38°C for 30 minutes. a,b: Different letters indicate significant differences between storage temperatures at a given storage time (P<0.05). An asterisk indicates a significant change after 72 h or 120 h storage as compared to 24 h for a given storage temperature (P<0.05).
