# Supplementary material for: Temperature limits for storage of extended boar semen from the perspective of the sperm's energy status
Source: Front Vet Sci. 2022 Aug 5;9:953021. doi: 10.3389/fvets.2022.953021 (PMC9388907; doi:10.3389/fvets.2022.953021)
Supplement: Supplementary file 1 [file Data_Sheet_1.zip › Supplemental Figure 2.docx]

after 15 minutes at room temperature

after 30 minutes at 38°C

**Supplemental Figure 2.** Viability and acrosome integrity.

The percentage viable, acrosome intact spermatozoa (propidium iodide & PNA-FITC negative) after 15 min incubation at room temperature or 30 minutes incubation at 38°C for boar semen samples stored at various temperatures in Beltsville Thawing Solution (BTS). Data are presented as means ± standard deviation (n= 7 boars). Different letters (a-e) indicate significant differences between storage temperatures and assessment times/temperatures (P< 0.05).
