# Supplementary material for: Temperature limits for storage of extended boar semen from the perspective of the sperm's energy status
Source: Front Vet Sci. 2022 Aug 5;9:953021. doi: 10.3389/fvets.2022.953021 (PMC9388907; doi:10.3389/fvets.2022.953021)
Supplement: Supplementary file 1 [file Data_Sheet_1.zip › Supplemental Table 1.docx]

**Supplemental Table 1.** Nucleotide concentrations in semen extender at different semen storage temperatures and after different storage times.

|  |  | ATP  (pmol/25 µL) | ADP  (pmol/25 µL) | AMP  (pmol/25 µL) |
| --- | --- | --- | --- | --- |
| 24 h | 5°C | 7 ± 7 | 61 ± 38 | 247 ± 81 |
|  | 10°C | 1 ± 2 | 14 ± 9 | 83 ± 35 |
|  | 17°C | 0 ± 0 | 2 ± 3 | 12 ± 7 |
|  | 25°C | 0 ± 0 | 1 ± 1 | 7 ± 5 |
|  |  |  |  |  |
| 72 h | 5°C | 2 ± 2 | 33 ± 10 | 264 ± 102 |
|  | 10°C | 1 ± 1 | 17 ± 10 | 76 ± 22 |
|  | 17°C | 0 ± 0 | 1 ± 2 | 11 ± 6 |
|  | 25°C | 0 ± 0 | 0 ± 0 | 8 ± 8 |
|  |  |  |  |  |
| 120 h | 5°C | 0 ± 1 | 14 ± 6 | 228 ± 110 |
|  | 10°C | 1 ± 1 | 7 ± 3 | 51 ± 19 |
|  | 17°C | 0 ± 0 | 1 ± 1 | 13 ± 6 |
|  | 25°C | 0 ± 0 | 1 ± 1 | 8 ± 5 |
