# Supplementary material for: Temperature limits for storage of extended boar semen from the perspective of the sperm's energy status
Source: Front Vet Sci. 2022 Aug 5;9:953021. doi: 10.3389/fvets.2022.953021 (PMC9388907; doi:10.3389/fvets.2022.953021)
Supplement: Supplementary file 1 [file Data_Sheet_1.zip › Supplemental Table 2.docx]

**Supplemental Table 2.** Motility parameters from computer-assisted semen analysis.

All values are mean and standard deviation. Different letters (A - C) in each column show significant differences between storage times (P < 0.05; n = 7 boars).

|  |  | Storage temperature | | | |
| --- | --- | --- | --- | --- | --- |
|  | Storage | 25°C | 17°C | 10°C | 5°C |
| TM (%) | 24 h | 88.3 ± 2.5^A^ | 87.9 ± 3.3^A^ | 79.6 ± 3.7^A^ | 55.3 ± 2.9^A^ |
|  | 72 h | 87.7 ± 2.6^A^ | 88.1 ± 3.4^A^ | 80.8 ± 3.4^A^ | 51.5 ± 2.4^B^ |
|  | 120 h | 86.6 ± 3.3^A^ | 87.1 ± 3.2^A^ | 78.8 ± 4.4^A^ | 47.3 ± 2.6^C^ |
|  |  |  |  |  |  |
| PM (%) | 24 h | 78.1 ± 4.9^A^ | 77.5 ± 5.0^A^ | 70.7 ± 3.8^A^ | 40.0 ± 1.9^A^ |
|  | 72 h | 78.2 ± 4.2^A^ | 76.4 ± 3.1^A^ | 68.7 ± 5.4^A^ | 36.0 ± 3.1^A^ |
|  | 120 h | 77.8 ± 3.8^A^ | 77.7 ± 2.6^A^ | 68.1 ± 3.6^A^ | 36.1 ± 1.7^A^ |
|  |  |  |  |  |  |
| VAP (μm/s) | 24 h | 62.8 ± 4.4^A^ | 63.0 ± 7.4^A^ | 64.5 ± 11.5^A^ | 62.2 ± 8.7^A^ |
|  | 72 h | 62.5 ± 5.6^A^ | 66.9 ± 9.5^A^ | 64.7 ± 13.2^A^ | 59.3 ± 8.8^A^ |
|  | 120 h | 54.8 ±8.2^A^ | 64.7 ± 7.6^A^ | 60.8 ± 12.1^A^ | 55.1 ± 14.7^A^ |
|  |  |  |  |  |  |
| VCL (μm/s) | 24 h | 85.2 ± 7.8^A^ | 91.2 ± 12.3^A^ | 82.3 ± 7.7^A^ | 82.9 ± 9.4^A^ |
|  | 72 h | 88.2 ± 10.5^A^ | 96.7 ± 11.3^A^ | 103.9 ± 12.5^B^ | 87.7 ± 9.5^B^ |
|  | 120 h | 82.0 ± 13.2^A^ | 95.1 ± 14.5^A^ | 96.8 ± 13.6^C^ | 80.1 ± 13.3^A,B^ |
|  |  |  |  |  |  |
| VSL (μm/s) | 24 h | 54.1 ± 4.8^A^ | 51.9 ± 7.7^A^ | 51.8 ± 11.9^A^ | 48.9 ± 7.6^A^ |
|  | 72 h | 53.4 ± 5.8^A^ | 55.0 ± 9.5^A^ | 49.8 ± 14.5^A^ | 47.0 ± 10.4^A^ |
|  | 120 h | 46.0 ± 7.7^A^ | 53.4 ± 7.0^A^ | 45.1 ± 10.9^A^ | 44.2 ± 13.3^A^ |
|  |  |  |  |  |  |

|  |  | Storage temperature | | | |
| --- | --- | --- | --- | --- | --- |
|  | storage | 25°C | 17°C | 10°C | 5°C |
| STR | 24 h | 0.86 ± 0.03^A^ | 0.82 ± 0.04^A^ | 0.79 ± 0.07^A^ | 0.78 ± 0.06^A^ |
|  | 72 h | 0.85 ± 0.04^A^ | 0.82 ± 0.04^A^ | 0.78 ± 0.11^A^ | 0.78 ± 0.08^A^ |
|  | 120 h | 0.83 ± 0.03^A^ | 0.82 ± 0.04^A^ | 0.73 ± 0.08^A^ | 0.79 ± 0.05^A^ |
|  |  |  |  |  |  |
| LIN | 24 h | 0.62 ± 0.05^A^ | 0.57 ± 0.06^A^ | 0.58 ± 0.07^A^ | 0.55 ± 0.04^A^ |
|  | 72 h | 0.58 ± 0.07^A^ | 0.56 ± 0.04^A^ | 0.45 ± 0.06^B^ | 0.55 ± 0.06^A^ |
|  | 120 h | 0.56 ± 0.06^A^ | 0.56 ± 0.08^A^ | 0.48 ± 0.07^A,B^ | 0.53 ± 0.08^A^ |
|  |  |  |  |  |  |
| WOB | 24 h | 0.73 ± 0.03^A^ | 0.69 ± 0.06^A^ | 0.69 ± 0.07^A^ | 0.68 ± 0.06^A^ |
|  | 72 h | 0.71 ± 0.05^A^ | 0.69 ± 0.06^A^ | 0.66 ± 0.1^A^ | 0.67 ± 0.08^A^ |
|  | 120 h | 0.67 ± 0.06^A^ | 0.68 ± 0.07^A^ | 0.63 ± 0.08^A^ | 0.67 ± 0.07^A^ |
|  |  |  |  |  |  |
| ALH (µm) | 24 h | 1.9 ± 0.3^A^ | 2.0 ± 0.3^A^ | 2.0 ± 0.4^A^ | 2.0 ± 0.2^A^ |
|  | 72 h | 2.0 ± 0.3^A^ | 2.1 ± 0.4^A^ | 2.4 ± 0.4^A^ | 2.1 ± 0.3^A^ |
|  | 120 h | 1.9 ± 0.2^A^ | 2.1 ± 0.3^A^ | 2.2 ± 0.3^A^ | 1.9 ± 0.4^A^ |
|  |  |  |  |  |  |
| BCF (Hz) | 24 h | 35.9 ± 2.3^A^ | 37.1 ± 2.1^A^ | 36.9 ± 2.3^A^ | 34.3 ± 2.0^A^ |
|  | 72 h | 37.8 ± 1.4^A^ | 38.3 ± 3.8^A^ | 36.6 ± 3.5^A^ | 31.5 ± 4.5^A^ |
|  | 120 h | 34.3 ± 4.1^A^ | 37.9 ± 2.7^A^ | 37.8 ± 3.0^A^ | 31.3 ± 4.0^A^ |
|  |  |  |  |  |  |

TM: Total motility PM: Progressive motility

VAP = average path velocity VCL = curvilinear velocity

VSL = straight-line velocity STR = straightness

LIN = linearity WOB = wobble

ALH = amplitude of lateral head-displacement BCF = beat cross frequency
